# Secondary Osteoporosis: A Still Neglected Condition

**DOI:** 10.3390/ijms24108558

**Published:** 2023-05-10

**Authors:** Vittoria Favero, Cristina Eller-Vainicher, Iacopo Chiodini

**Affiliations:** 1 Department of Biotechnology and Translational Medicine, University of Milan, 20122 Milan, Italy; vittoria.favero@unimi.it; 2 Fondazione IRCCS, Cà Granda Ospedale Maggiore Policlinico, 20122 Milan, Italy; cristina.eller@policlinico.mi.it; 3 Ospedale Niguarda Cà Granda, Piazza Ospedale Maggiore 3, 20161 Milan, Italy

The condition of “secondary osteoporosis” is defined as a bone loss that results from specific well-defined clinical disorders [1]. Among patients with osteoporosis, up to 30% and 60% of female and male subjects are actually affected by a secondary cause of bone fragility [2]. Individuating an underlying disease that causes bone fragility could help to avoid erroneous and potentially deleterious treatments. Furthermore, treating the specific cause of osteoporosis could, in some cases, not only cure osteoporosis itself, but also reduce the extra-skeletal complications of the underlying disorders. Very often indeed, bone fragility fractures are the manifest symptoms of diseases that otherwise could remain concealed for many years. This is, for example, the case of mild cortisol excess, in which an unexplainable bone fragility may be the first symptom of a hidden hypercortisolism, which is known to be associated with increased mortality due to cardiovascular and infectious consequences [3]. Importantly, by removing the cause of cortisol excess, it is possible to both decrease fracture risk and ameliorate cardiometabolic risk [4].

Regarding glucocorticoid-induced osteoporosis, in the Special Issue on Osteoporosis 2.0, Martin-Aragon and co-authors investigated the possible antiapoptotic activity in glucocorticoid-treated osteoblastic cells of a bovine colostrum that is known to exert a neuroprotective effect against glucocorticoid-induced neuronal damage. In osteoblasts, colostrum prevented the decrease in cell viability and the increase in oxidative stress caused by glucocorticoid exposure, supporting the hypothesis that bovine colostrum, as a complex and multi-component dairy product, in addition to its neuroprotective action, may influence osteoblastic cell survival. The mechanisms through which colostrum may exert this effect are related to the potential of IGF-1 (a colostrum constituent) in activating PI3K/Akt and MAPK/ERK signaling pathways, the inhibition of which is associated with dexamethasone-induced cell death. Moreover, a remarkable component of bovine colostrum is colostrinin, a mixture of at least 32 proline-rich polypeptides, which has been shown to modulate intracellular ROS levels in cultured cells via the regulation of glutathione metabolism, antioxidant enzyme activity, and mitochondria function [5].

It is well known in the clinical practice that, besides glucocorticoids, which are known to reduce bone quality, other drugs have this negative potential [6,7]. Among potentially bone-damaging drugs conventional antiepileptics have been demonstrated to be associated with bone structure abnormalities. In this Special Issue, Matuszewska and co-workers report the results of a study on the effect of stiripentol on rat bones. They found a reduction in bone apposition and in calcitriol levels in rats treated with stiripentol than in controls and, of importance, lower bone volume fraction, lower trabecular thickness, higher trabecular pattern factor, and a higher structure model index, as evaluated by the micro X-ray computed tomography of the tibias in the stiripentol group. The authors speculated that, besides the possible negative effects of low calcitriol levels on bone microarchitecture, a direct impact of STP on bone cells cannot be excluded. Indeed, stiripentol may induce allosteric changes in the GABAA receptors and modulate GABAergic transmission. Since GABAA and GABAB receptors have been identified in chondrocytes in the growth plate and some data suggest that GABA can regulate the state of the growth plate in an autocrine/paracrine way, it can be hypothesized that the negative effects of stiripentol on bone microarchitecture may be related to its influence on GABA transmission [8]. This study opens up two different research fields. On the one hand, based on the results of this experiment on rats, further prospective human studies are probably worth being carried out. On the other hand, GABA transmission could be seen as a possible target for studying the pathophysiologic mechanisms for maintaining bone quality.

Dissecting the pathogenetic pathways and even the genetic background of the forms of secondary osteoporosis also gives us the opportunity to find out new potential targets for curing, even for the so-called primary osteoporosis. In this regard, the Special Issue, Secondary Osteoporosis 2.0, includes a thorough review of the clinical bone phenotypes and the associated bone fragility in rare congenital metabolic bone disorders, following a disease taxonomic classification based on deranged bone metabolic activity. Indeed, to date, more than 100 different Mendelian-inherited bone diseases have been described, resulting from mutations in more than 80 different genes involved in the regulation of bone and mineral metabolism. Importantly, these disorders affect bone strength by either increasing or decreasing bone formation or bone resorption. Genetic causes of low bone formation are inactivating mutations in genes that are necessary for successful osteoblast differentiation, such as LRP5, RUNX2, SP7, and NOTCH2, and genes that regulate osteoblast-driven mineralization, such as IFITM5 and PLS3. On the contrary, an enhanced number of, and/or the enhanced activity of, osteoblasts can result in the increased deposition of mineralized bone and increased bone density. These diseases are mainly caused by mutations in genes (RUNX2, LRP5, AMER1, and LEMD3) that regulate the differentiation of mesenchymal precursors toward the osteoblastic lineage, or in genes that influence osteoblastic activity (SOST). Despite increased bone density, these disorders result in bone fragility due to reduced bone quality. Genetic diseases associated with reduced bone resorption cause a decrease in osteoclast number and/or function, due to germline mutations in genes that regulate either osteoclast differentiation (TNFRSF11A and TNFSF11) or osteoclast activity (CA2, CLCN7, and CTSK). Under these conditions, the reduced ability of repairing the micro-cracks confers a decreased bone strength, favoring bone fragility. Again, a reduction in bone quality, rather than in bone density, is the main cause of the increased fracture risk under these conditions. An important element of bone quality is represented by collagen. Genetic defects may affect the collagen type 1 synthesis and structure, the post-translational collagen modifications, and the processing and crosslink of collagen. Other genetic alterations may influence the bone microenvironmental regulators (i.e., alkaline phosphatase), bone-regulating cytokines (i.e., RANK/RANKL/OPG system), and crucial pathways for the cross-talk of bone cells (i.e., LRP5-Wnt signaling and bone morphogenetic protein receptors). Finally, bone fragility could result from an altered activity of calciotropic and phosphotropic hormones due to genetic defects, like in disorders due to an alteration in parathormone signaling (i.e., pseudohypoparathyroidism) in disorders due to altered vitamin D metabolism and activity, as well as in congenital disorders of phosphate homeostasis. Studying the genetic basis of some forms of secondary osteoporosis allows us to understand that a typical characteristic of these forms is an alteration in bone quality which increases the fracture risk, even in the absence of a severely reduced bone mineral density. Unfortunately, to date, estimating bone quality remains a challenge in route clinical practice and further studies are needed to transform this scientific evidence in practical tools for curing our patients [6].

In recent years, a large amount of data has been produced, suggesting that bone health is strictly associated with muscle health and that bone and muscle should be considered a single endocrine unit [9]. Among the various factors responsible for the crosstalk between bone and muscle and for the endocrine effects of the bone and muscle unit, irisin has emerged as a very promising molecule [10]. In this Special Issue, Colucci and collaborators explore the possible role of irisin for accelerating bone fracture healing. They subjected 8-week-old male mice to closed, transverse, mid-diaphyseal tibial fractures and treated them with intraperitoneal injection of a vehicle or irisin immediately following fracture for 10 days or 28 days. They found that, at 10 days, irisin accelerated the transition of cartilage callus into bony callus, as shown by the important increase in collagen type X, RUNX2 levels and osteoclasts number and by the reduced content of proteoglycans and of SOX9 expression. Importantly, 28 days after fracture, the total callus volume, the bone volume, and the bone mineral content were increased by about 70% in irisin-treated mice than in controls, as demonstrated by microCT analyses. Again, this study opens up research frontiers on both the possible application of irisin for accelerating bone healing in humans and the possible development of studies aimed at dissecting the molecular pathways by which irisin affects bone reconstruction after a fracture [11].

The idea that bone could be a target tissue in many systemic disorders has fostered the evaluation of bone health in patients affected by disorders previously not known to be associated with bone consequences. In this Special Issue on Secondary Osteoporosis 2.0, Chen and co-workers reviewed recent evidence on the mechanisms of bone metabolism alterations in chronic inflammatory arthritis. In particular, new data have been produced on the role of some important pathways and factors such as the receptor activator of the nuclear factor-κB ligand, anti-citrullinated protein antibodies, Wnt signaling and Dickkopf-related protein 1, the interleukin-17/23 axis, the Janus kinase, and the signal transducer and activator of transcription signaling in affecting bone health in patients with chronic inflammatory arthritis. These findings represent the pathophysiologic basis of the possible positive effects of drugs on bones, as tumor necrosis factor inhibitors, abatacept, rituximab, tocilizumab, Janus kinase inhibitors, and inhibitors of the interleukin-17/23 axis are discussed [12]. This evidence supports the idea that bones have a high sensitivity to potentially all types of disorders that could affect our organisms, particularly inflammatory ones. Consequently, clinicians should consider evaluating bone health in all patients affected by chronic inflammatory conditions.

Gastro-intestinal diseases do not represent an exception due to both a chronic inflammatory status and altered nutrients absorption. Despite their wide prevalence in the general population, the skeletal implications of many gastrointestinal diseases are often underestimated in clinical practice. Merlotti and coauthors have reviewed the available literature data on the role of major gastrointestinal disorders in the pathogenesis of osteoporosis and fragility fractures. Importantly, not only are the severe gastrointestinal disorders considered to be important factors that contribute to bone damage, but even apparently less severe conditions, such as the helicobacter pylori infection, are thought to be potentially deleterious. In addition, the intestinal microbiome, regulating gastrointestinal permeability, has been found to exert a relevant role in triggering the inflammatory pathways that are critical for osteoclast activation during estrogen deficiency. The consequence of these data in clinical practice may be that using probiotics might be beneficial as a therapeutic approach for postmenopausal osteoporosis, and short-term trials seem to confirm this hypothesis [13]. Thus, knowledge on the mechanisms by which gastrointestinal disorders may negatively impact bone health is of utmost importance in order to avoid wrong treatment and optimize the effects of the needed drugs, even with the use of probiotics and/or avoiding potentially dangerous diets [14].

An important awareness that has been spreading in recent years is that osteoporosis is not a woman-related disease, but that it could affect even men. However, as nicely pointed out by Vescini and collaborators in this Special Issue, male osteoporosis is still a largely underdiagnosed pathological condition. Indeed, although the prevalence of osteoporosis in women is higher than in men, up to 40% of overall osteoporotic fractures affect men. On top of that, in males, the mortality and morbidity related to hip fractures are twice as high in men compared to women. Several factors influence bone health in men, such as the increased levels of follicle-stimulating hormones levels and/or decreased levels of testosterone and estrogen. These factors interact with the genetic background in determining the peak of bone mass, the maintenance of bone density during the adult life, and its decline during ageing. Since men should be at lower risk of osteoporosis and bone fragility than women, because males have wider bones than females, the finding of osteoporosis or of bone fragility in a male patient should always push the physician to rule out a cause of secondary osteoporosis. Moreover, since fracture-related mortality is importantly increased in men, treating male subjects with osteoporosis may impact on mortality even more than in women [15].

In conclusion, further research should focus more on the mechanisms that underly the negative effect of chronic disorders on bones. While waiting for the advances in bone research, physicians should always consider that an unexplainable bone fragility could be the sole and the presenting symptom of an otherwise silent disease, and that in patients with a known chronic disorder, bone health should be evaluated for the other target organs of our body.
